# Microbial assay of N_2_ fixation rate, a simple alternate for acetylene reduction assay

**DOI:** 10.1016/j.mex.2017.11.010

**Published:** 2018-01-06

**Authors:** Subhajit Das, Tarun Kumar De

**Affiliations:** Department of Marine Science, University of Calcutta, 35, B.C. Road, Calcutta, 700 019, India

**Keywords:** Acetylene reduction assay, N_2_-fixing bacteria, Microbial bio-assay, Sundarban ecosystem

## Abstract

Nitrogen is an essential element for living creatures in every ecosystem but nitrogen cannot be absorbed by the plant itself directly from the atmosphere, so for nitrogen, plant depends on both free living and symbiotic microbes present in the soil. Nitrogen fixation potentiality of the soil thus reveals its fertility with respect to nitrogen. Researchers developed and modified techniques for measuring nitrogen fixation rate of the soil and acetylene reduction assay (ARA) technique became the most popular till now. At the same time this technique has few limitations especially for the researchers from third world country due to lack of special infrastructure in the laboratory and the most required instrument for this technique, gas chromatograph machine, is very expensive. Any alternation of this technique is deserved highly for the researchers from the developing countries. The present work/method explained a new approach for determination of nitrogen fixation rate and this new method was named as “Microbial bio-assay”. In this technique nitrogen fixers were cultured in specific medium and condition and after required time of interval the amount of nitrogen fixed by them were calculated. Exploration of soil of the Sundarban mangrove ecosystem was performed regarding the microbial N_2_ fixing capacity of that particular ecosystem.

• The nitrogen fixation rate measured by acetylene reduction assay (ARA) was 1.13 times lower than the N_2_ fixation rate measured by microbial bio-assay.
• Microbial bio-assay can be used as an alternate of ARA method to measure N_2_ fixation rate. The rates of N_2_ fixation measured by both two methods were positively correlated with the population of N_2_ fixing bacteria present in the soil of that particular ecosystem (R^2^ = 0.85, p < 0.005, n = 85 for microbial bio-assay and R^2^ = 0.78, p < 0.005, n = 85 for ARA).

## Graphical abstract

Comparison between ARA and Microbial bio-assay in terms of N2 fixation rate.

**Figure:**
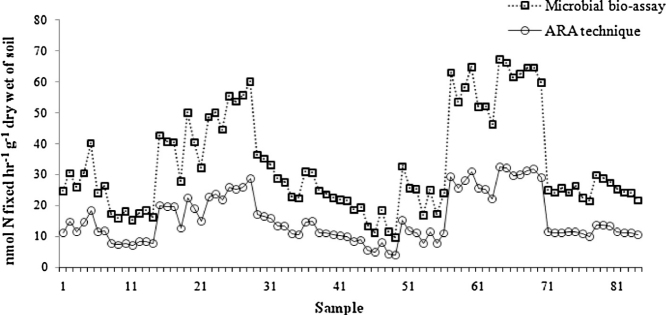

## Introduction

Atmospheric nitrogen can be fixed either by free living nitrogen fixing diazotrophs or by symbiotic microbes associated with plant roots. Nitrogen fixing bacteria plays the vital role in the biological conversion of dinitrogen (N_2_) gas into ammonia (NH_3_), the principal route of biological nitrogen fixation. The abundance and the ability of the N_2_ fixation rate of nitrogen fixing bacteria are essential to explore for understanding the ecological status of an environment regarding the nitrogen cycle [1]. Quantification of N_2_ fixation by nitrogen fixers is necessary for knowing their contribution in total nitrogen budget in a particular ecosystem [2]. For quantification of N_2_ fixation rate researchers were mainly dependent on acetylene reduction assay which relies on the preferential reduction of acetylene (C_2_H_2_) to ethylene (C_2_H_4_) by nitrogenase, instead of reducing N_2_ to NH_3_ [3]. In addition to acetylene reduction assay microbial bio-assay can also be encouraged where applicable. In this new technique N_2_ fifing bacteria were grown in specific medium with required condition. After required interval of time the number of bacteria increased was counted. The total content of nitrogen present in the bacterial cell was measured before and after incubation period. the amount of nitrogen content increased was calculated as nitrogen fixed and by dividing it with incubation period, nitrogen fixation rate was calculated. The total population of nitrogen fixing bacteria along with the indirect (acetylene reduction assay) and direct (microbial bio-assay) quantification of nitrogen fixation rate can give a comparative profile of nitrogen budget of the ecosystem concerned. Nitrogenase enzyme required anoxic conditions to carry out N_2_ fixation. Thereby, Sundarban mangrove it is an appropriate ecosystem to compare N_2_ fixation methodologies. Previous studies showed that nitrogen fixation was accelerated in anoxic condition and it became necessary to explore the anoxic soil of Sundarban mangrove ecosystem for understanding the nitrogen fixation capacity of that soil relating with the population of nitrogen fixing bacteria. In the present work N_2_ fixation rate of the Sundarban mangrove ecosystem was measured both by direct and indirect method for a comparison study and the acceptability of the microbial bio-assay was monitored.

## Methodology

### Study site

**Fig. 1:**
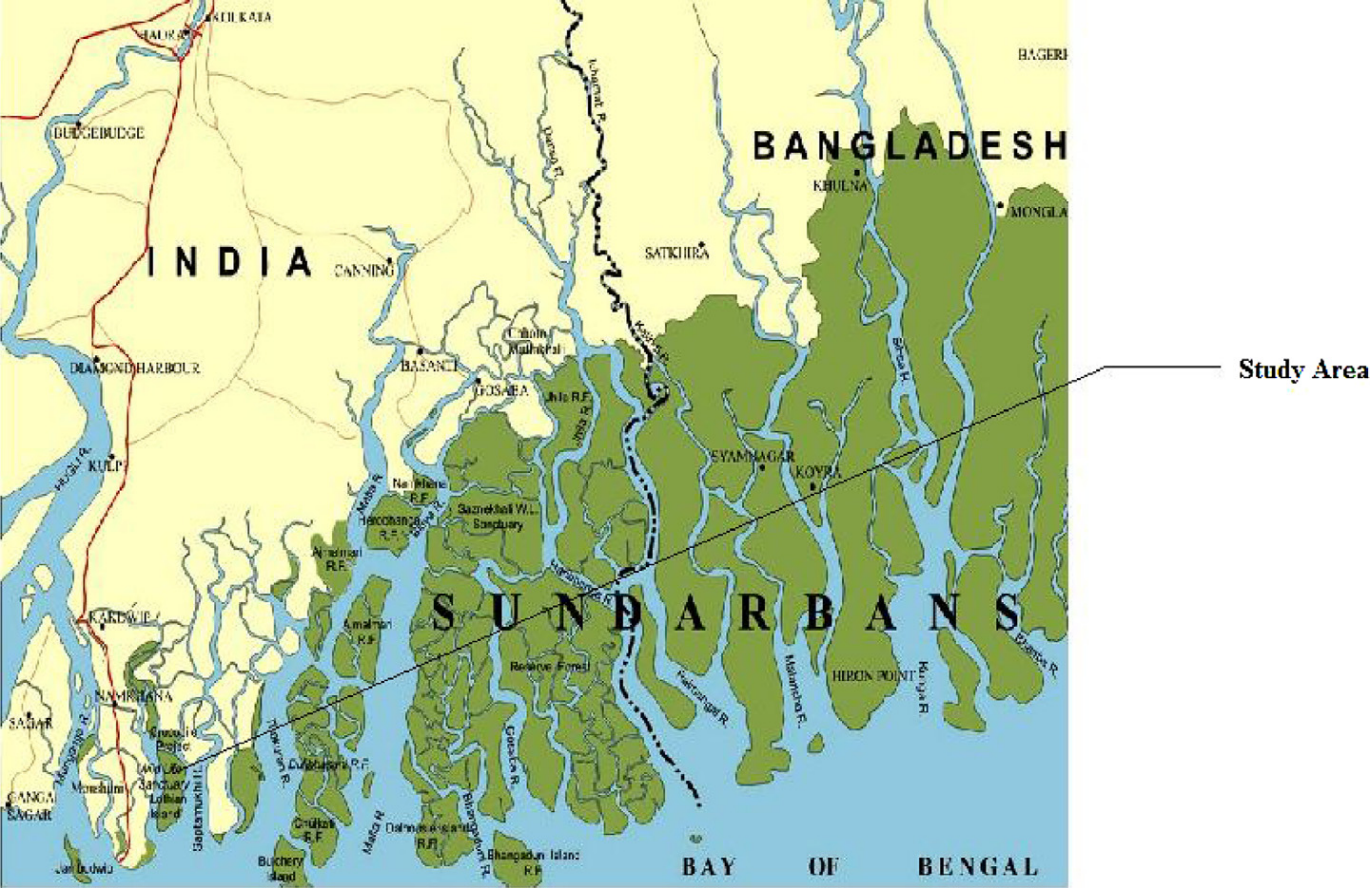
Map showing the study area.

Geographically in between 21°31′N and 22°30′N and longitude 88°10′E and 89°51′E the Sundarban Mangrove forest is located along the North East coast of Bay of Bengal, India. This mangrove forest is a part of the estuarine system of the River Ganges, NE coast of Bay of Bengal (Fig. 1), covering an area of 9630 km^2^. It constitutes several numbers of discrete islands. The climate in the region is characterized by the southwest monsoon (June–September), northeast monsoon or post-monsoon (October–January), and pre-monsoon (February–May); 70%–80% of annual rainfall occurs during the summer monsoon (southwest monsoon). The tide in this eastern complex is semidiurnal in nature with spring tide ranging between 4.27 m and 4.75 m and neap tide range between 1.83 m and 2.83 m. It is a unique bioclimatic zone in between the land and ocean boundaries of the Bay of Bengal and the largest delta on the globe. The deltaic terrain of Sundarban Biosphere Reserve comprises mainly saline alluvial soil consisting of clay, silt, fine and coarse sand particles.

### Description of sampling

Soil samples of 10 g were collected randomly from the deep forest region of Sundarban mangrove ecosystem from January to December with 5 cm segment from the surface to 60 cm of depth. Samples were transferred to the laboratory immediately in sterilized iced (4–6 °C) condition.

### Microbial bio-assay

• At first the soil sample collected from each depth was homogenized separately with the sterilized phosphate buffer isotonic solution.
• The soil suspension was then filtered through a membrane filter (pore size, 0.8 μm) using vacuum pump. This filtration was done to separate bacterial cells from other microbes and benthic phytoplankton as previous study reported that soil bacterial cells may attain maximum of 0.5 μm in diameter [4].
• The filtrate thus contained only bacterial cells. Serial dilution up to 10^−4^ was done using sterilized phosphate buffer isotonic solution and 1 ml of this dilution bacterial suspension was inoculated into 30 different petriplates containing 15 ml of nitrogen free medium, Jensen’s medium (Composition: Sucrose 20 g L^−1^, Dipotassium phosphate 1 g L^−1^, Magnessium sulphate 0.5 g L^−1^, Sodium Chloride 0.5 g L^−1^, Ferrous sulphate 0.1 g L^−1^, Sodium molybdate 0.005 g L^−1^, Calcium carbonate 2 g L^−1^, Agar 15 g L^−1^) [5].
• The plates were incubated for 2 weeks at 30 °C under N_2_ atmosphere of 5 ppmv of concentration in an isolated incubation chamber. Pure colonies were obtained by repeating subculture through streaking on Jensen’s medium.
• Morphologically different colonies were isolated and subcultured for 10 generations to be confirmed that the plates contained only free living N_2_ fixing bacteria. Pure culture obtained from the 10th generation was isolated from the medium with phosphate buffer saline solution.
• From this bacterial suspension serial dilution was made up to 10^−4^ using sterilized phosphate buffer isotonic solution and 1 ml of this solution was inoculated on petriplates contained 15 ml of Jensen’s medium and incubated under N_2_ atmosphere. This was done in duplicate. The petriplates were monitored in an interval of 12 h to count the increase in number of colony forming unit.

Total bacteria cell = cfu X number bacteria cell present in that colony

• Each colony was then monitored for quantification of free living N_2_ fixing bacteria by using haemocytometer.
• Total bacterial cell generated was calculated by multiplication of colony number and the bacterial cell found in the respective colony.
• From the replicate petriplates microbial sample was analyzed for quantification of nitrogen present in their cell by the high-temperature catalytic oxidation (HTCO) method [6].
• The amount of N_2_ fixed by the free living N_2_ fixing bacteria was divided by the incubation period for getting the rate of N_2_ fixation rate.

$$\text{Rate}\;\text{of}\;{\text{N}}_{2}\;\text{fixation}=\frac{{\left(\text{amount}\;\text{of}\;\text{N}\;\text{in}\;\text{bacterial}\;\text{cell}\right)}_{\text{after}\;2\;\text{weeks}}-{\left(\text{amount}\;\text{of}\;\text{N}\;\text{in}\;\text{bacterial}\;\text{cell}\right)}_{\text{Initial}}}{(\text{Incubation}\;\text{Period})\;\text{X}\;\text{mass}\;\text{of}\;\text{dry}\;\text{wt}\;\text{of}\;\text{soil}}$$

### Acetylene reduction assay

Soil slurry samples were collected with 5-mL sterile syringes with the top end of the syringe removed. Then, 10% of the atmosphere of each vial containing 10 ml soil slurry was substituted with gaseous acetylene and incubated at 28^∘^C. Ethylene production in each vial was quantified, using a gas chromatograph equipped with a flame ionization detector and a capillary column. The chromatograms were used to integrate the areas of the curves of C_2_H_2_ and ethylene (C_2_H_4_) to estimate C_2_H_4_ production [7].

### Quantification of N_2_ fixers

Homogenization of 10 g of soil samples from different depth was done in sterile phosphate buffer. Serial dilutions up to 10^−4^ were made and inoculation was done with 0.1 ml in the Jensen’s medium. Isotonic solution with the soil was prepared from NaCl and sterilized distilled water (1 ltr) and pH maintained at 8.3 [8].

### Statistical analysis

Multiple regression analysis was done using a MINITAB (version 13.0) statistical package.

## Result

**Fig. 2:**
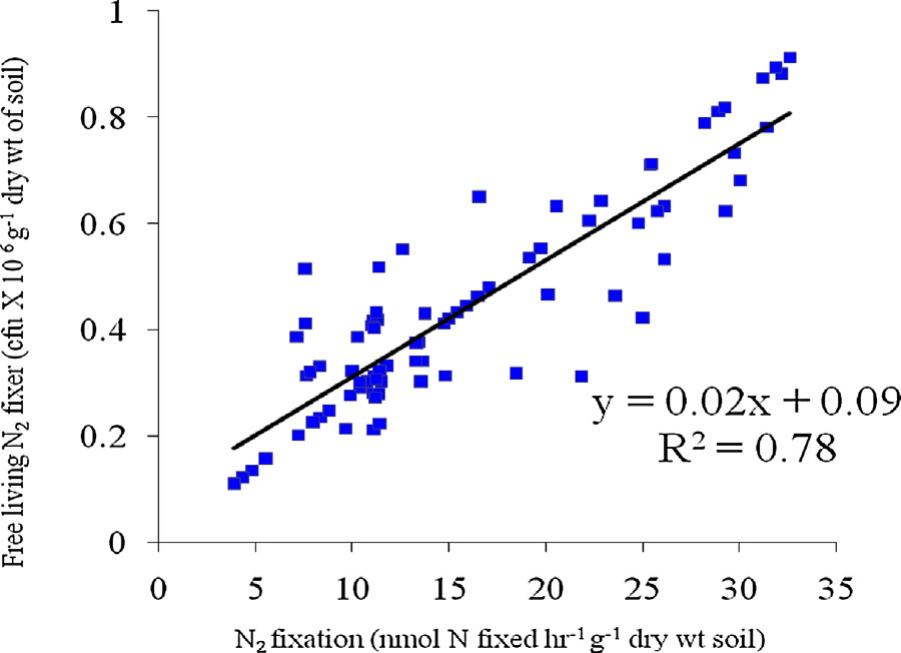
Correlation graph between N_2_ fixation rate by Acetylene Reduction Assay and population of free living N_2_ fixing bacteria.

**Fig. 3:**
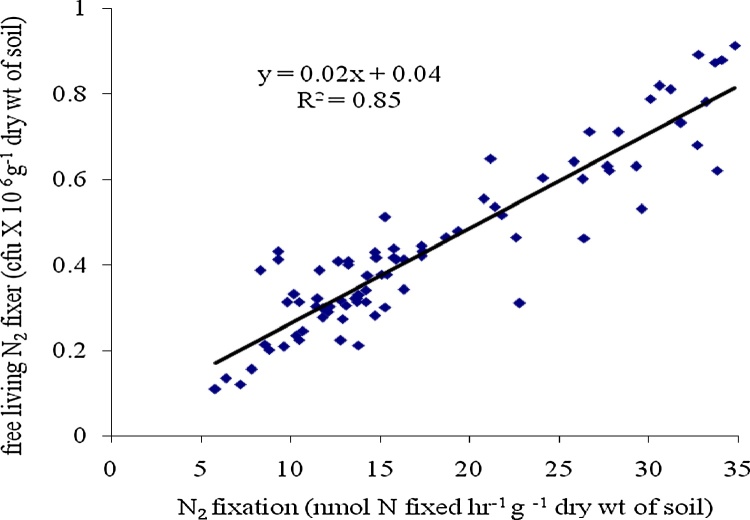
Correlation graph between N_2_ fixation rate by Microbial bio Assay and population of free living N_2_ fixing bacteria.

**Fig. 4:**
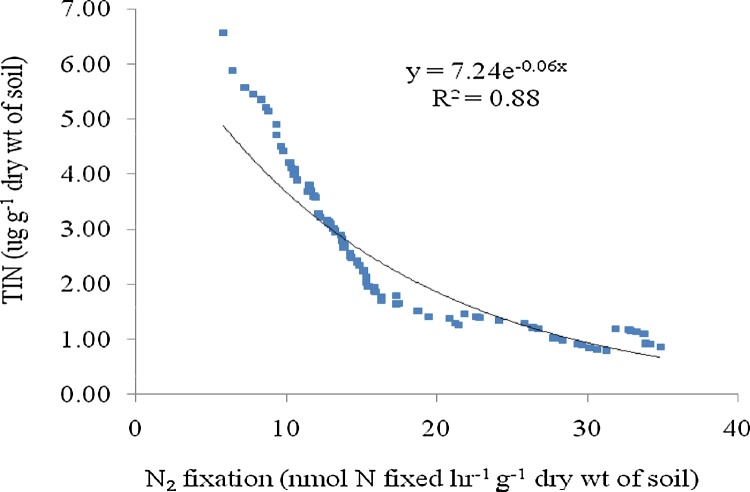
Correlation graph between N_2_ fixation rate by Microbial bio Assay and total inorganic nitrogen (TIN) present in the soil.

**Fig. 5:**
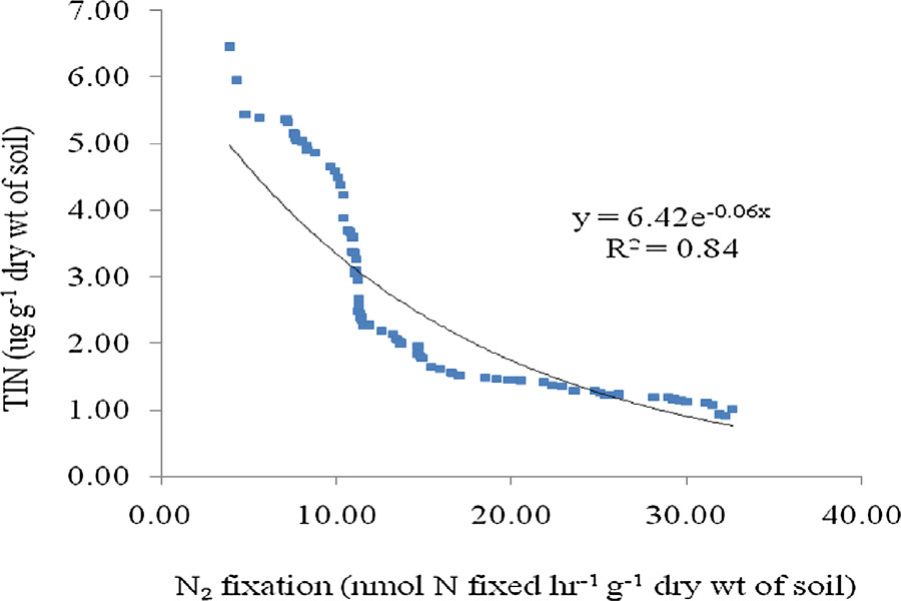
Correlation graph between N_2_ fixation rate by acetylene reduction assay (ARA) and total inorganic nitrogen (TIN) present in the soil.

The rate of nitrogen fixation measured by bio-assay showed positively correlation with the population of free living N_2_ fixing bacteria present in the soil (R^2^ = 0.85, p < 0.005, n = 85). The rate of nitrogen fixation measured by acetylene reduction assay (ARA) was found to be positively correlated with the population of free living N_2_ fixing bacteria (R^2^ = 0.78, p < 0.005, n = 85). Nitrogen fixation measured by bio-assay showed R^2^ value 1.08 times more than that of acetylene reduction assay (Fig. 2, Fig. 3). More R^2^ value suggested more acceptability of bio-assay technique over ARA method. Previous study reported the difficulties in ARA technique due to slow diffusion of acetylene and ethylene through soil slurries [9]. Total inorganic nitrogen (TIN) content of the soil was found to be negatively correlated with nitrogen fixation rate measured by acetylene reduction assay (R^2^ = 0.84, p < 0.005, n = 85) and nitrogen fixation rate measured by bio-assay (R^2^ = 0.88, p < 0.005, n = 85). The soil sample with less TIN showed more nitrogen fixation rate. The lower range of content of soil TIN (0.91 μg g^−1^ dry wt of soil) showed higher range of nitrogen fixation rate (32.18 nmol N fixed hr^−1^ g^−1^ dry wt of soil). The R^2^ value regarding it was 1.04 times more for the bio-assay method than that of ARA method (Fig. 4, Fig. 5).

## Discussion

The present study implied its effectiveness for measurement of N_2_ fixation rate in wetlands or swamps like Sundarban mangrove forest. Difficulties of ARA can be solved by this simple incubation technique where applicable. Similar technique can be applied for different strains of Nitrogen fixer culturing in different medium. The probable role of the composition of medium regarding the nitrogen fixation rate can be monitored by the newly approached assay. Quantitative and qualitative modifications in culture medium for microbial bio-assay should be correlated with the ARA technique in future and the best suited medium composition can be used for getting more exact results. The effect of the physico-chemical parameters of the nutrient medium on the rate of N_2_ fixation can be predicted by this bio-assay.
